# Seasonality Disrupted: Post-Pandemic Trends in Otorhinolaryngological Infections

**DOI:** 10.3390/jcm13185388

**Published:** 2024-09-12

**Authors:** Julia Pickert, Sarah Riemann, Andreas Spörlein, Andreas Knopf

**Affiliations:** Department of Otorhinolaryngology-Head and Neck Surgery, Faculty of Medicine, University of Freiburg, Klinik für Hals-Nasen-Ohrenheilkunde, Kopf- und Halschirurgie, Killlianstraße 5, 79106 Freiburg, Germanysarah.riemann@uniklinik-freiburg.de (S.R.); andreas.spoerlein@uniklinik-freiburg.de (A.S.)

**Keywords:** COVID-19, otorhinolaryngological infections, non-pharmaceutical interventions

## Abstract

**Background:** The COVID-19 pandemic has notably affected the epidemiology of various infectious diseases. The imposed public health measures and disruptions in vaccination programs have potentially altered the patterns of these diseases post pandemic. **Objective:** To investigate the change in epidemiology of otorhinolaryngological infectious diseases in adult and pediatric patients after the COVID-19 pandemic and the relaxation of public health measures. **Methods:** A retrospective cohort study was conducted at a large tertiary university otolaryngology department in the south of Germany, examining admissions with specific ICD-10 diagnoses from 2019 to 2023. Data were seasonally categorized and statistically analyzed. **Results:** A total of 1728 inpatient cases were analyzed. There was a significant increase in otorhinolaryngological infections in the post-pandemic winter of 2022, particularly of peritonsillar abscesses, acute tonsillitis and acute mastoiditis. No significant post-pandemic spike in mononucleosis was observed. The duration of hospitalization was shorter in 2022, and the median age of patients did not change significantly pre- versus post-pandemic. **Conclusions:** The study indicates a significant post-pandemic rise in otorhinolaryngological infections. Remarkably, the typical “dip” in infections during the summer months was not observed in the post pandemic years, possibly reflecting the impact of the termination of non-pharmaceutical interventions. Mononucleosis was the only infection not following this pattern.

## 1. Introduction

The COVID-19 pandemic has influenced the epidemiology of many other diseases worldwide. It disrupted pediatric vaccination programs in numerous countries, reduced hospital admissions for non-COVID-19 infections and changed the seasonality of some diseases. The World Health Organization reported the most significant decline in children receiving vaccinations in the last 30 years [1]. Poliomyelitis reemerged in previously polio-free declared countries in 2022 and in several countries, for example France, vaccine delivery was notably decreased at least until 2021 [2,3].

Hospital admissions for all non-COVID-19 infections were significantly lower during the lockdowns in Denmark and significantly less hospitalized patients with acute respiratory diseases other than COVID-19 were reported in Brazil [4,5]. Admissions for pediatric upper respiratory tract infections including acute otitis media in children up to the age of 9 years were also found to be reduced [6]. The usually observed seasonal peak in acute pediatric mastoiditis in winter failed to appear during the COVID-19 pandemic in the winters of 2020 and 2021 in Britain [7].

Not just the number of hospital admissions was decreased during the initial months of the COVID-19 pandemic, but also the number of critical procedures in otorhinolaryngological departments including emergency surgery for acute inflammatory diseases, such as acute mastoiditis and neck abscesses [8]. The numbers of tonsil surgeries in Germany were reduced during and after the lockdown in 2020 and continued to remain below pre-pandemic levels until at least September 2021 [9].

The seasonalities of the influenza virus, the human respiratory syncytial virus (RSV) and of human corona viruses other than SARS-CoV-2 are widely recognized [10,11]. However, after the pandemic, we saw out-of-season RSV epidemics in several countries [12,13]. Moreover, post-pandemic changes in the epidemiology of infections in children have been observed [14]. In 2022, the United Kingdom noted an unusual increase in cases of tonsillitis and scarlet fever caused by *Streptococcus pyogenes* [14]. Moreover, compared to the pre-pandemic years of 2017 to 2019 there was an increase of invasive group A streptococcal disease in children up to ten years of age [14].

This study aims to assess whether there has been a change in the epidemiology of otorhinolaryngological infectious diseases in adult and pediatric patients after the COVID-19 pandemic and the easing of public health measures. We hypothesized that the number of otorhinolaryngological infectious diseases has risen in the post-pandemic winter 2022.

## 2. Methods

We conducted a retrospective cohort study of the years 2019–2023 and analyzed admissions to the department of otorhinolaryngology, head and neck surgery, at the university hospital of Freiburg. We examined the most common otorhinolaryngological infectious diseases and included patients with the following: ICD-10 (International Classification of Diseases, Tenth Revision) diagnosis codes: acute tonsillitis (J03.0-9), peritonsillar abscess (J36), mononucleosis (B27.0-9), acute pharyngitis (J02.0-9), acute laryngotracheitis (J04.0-2), acute sinusitis (J01.0-9) and acute mastoiditis (H70.0-9) with the need for inpatient treatment.

All peritonsillar abscesses and acute mastoiditis were admitted. The admittance policies for the other diagnoses were insufficient symptom control, for example aphagia or dyspnea, with exhaustion of conservative measures such as oral antibiotics and analgesia. We have chosen this representative selection of diseases because they cover a broad spectrum of otorhinolaryngological infectious diseases. Furthermore, we only wanted to include ICD-10 diagnosis codes that occurred in considerable numbers and might impact clinical practice post pandemic. Therefore, we focused our in-depth analysis on the four most frequent infections.

In order to better reflect the seasonal fluctuations of otorhinolaryngological infectious diseases, we decided to display our data in seasons rather than months or years. Spring was previously described as the season with the most acute otorhinolaryngological infections [15]. The seasons were derived meteorologically for the northern hemisphere, with spring defined from March to May, summer from June to August, fall from September to November and winter from December to February of the following year [16].

Statistical analysis of the data obtained was performed with the Statistical Package for the Social Sciences (SPSS Inc., Chicago, IL, USA). Continuous variables were represented by the arithmetic mean and the corresponding standard deviation. Categorical variables were represented by absolute and relative frequencies. Group comparisons of categorical variables were performed using the chi-square test assessing whether there is a significant association between two categorical variables in our data or, for small data sets, the Fisher exact test. For continuous variables, the unpaired Student’s t-test was applied. Differences between more than two groups were calculated by ANOVA (Analysis of Variance) and subsequent post-hoc analyses. A *p*-value of <0.05 was defined as significant in all statistical tests.

## 3. Results

We analyzed the data of all 1728 inpatients presenting with one of the previously defined otorhinolaryngological infectious diseases between 1 March 2019 and 28 February 2023. In total, 918 patients exhibited a peritonsillar abscess, 316 patients had an acute tonsillitis, 162 had mononucleosis, 135 presented with an acute mastoiditis, 107 an acute sinusitis, 69 an acute laryngotracheitis and 21 an acute pharyngitis. An in-depth analysis was focused on the four most frequent infections.

### 3.1. Significant Increase in Otorhinolaryngological Infections in the Post-Pandemic Winter

**Figure 1:**
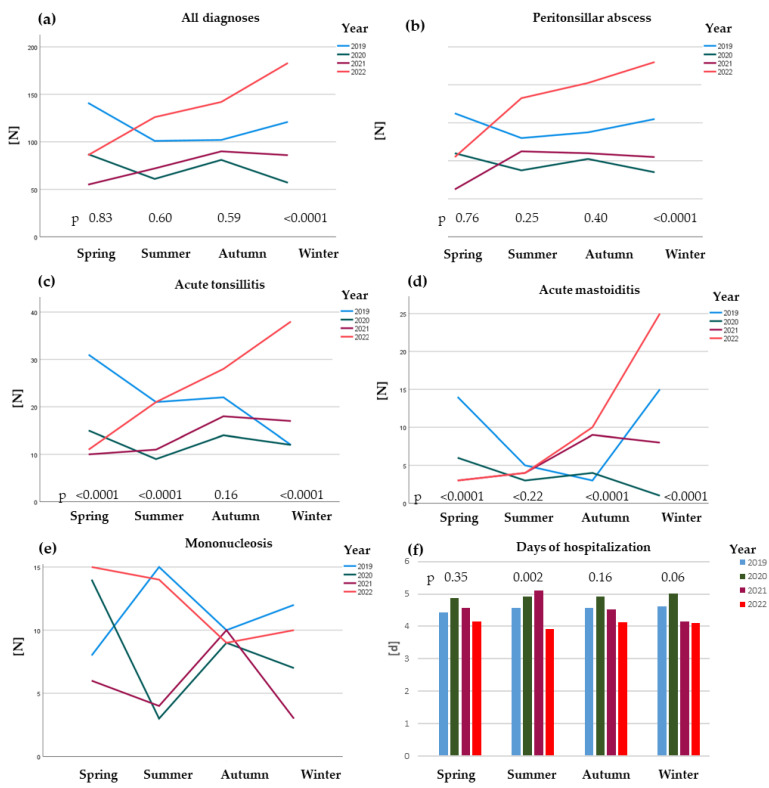
(**a**) The number of inpatients with the above defined otorhinolaryngological infections [N] per season over the course of the years 2019–2022. (**b**) The number of inpatients with peritonsillar abscesses [N] per season over the course of the years 2019–2022. (**c**) The number of inpatients with acute tonsillitis [N] per season over the course of the years 2019–2022. (**d**) The number of inpatients with acute mastoiditis [N] per season over the course of the years 2019–2022. (**e**) The number of inpatients with mononucleosis [N] per season over the course of the years 2019–2022. *p*-values were not significant and are therefore not displayed. (**f**) Days [d] of hospitalization across all diagnosis groups per season during the years 2019–2022.

We compared the number of inpatients with otorhinolaryngological infections per season over the course of the years 2019–2022. In spring 2019, we saw a high level of otorhinolaryngological infections, followed by a seasonal reduction in summer and autumn 2019 and a renewed increase in winter. This reflects the usual seasonal pattern. Corresponding to the beginning of the COVID-19 pandemic, the German government implemented the first lockdown in March 2020 [17], the number of otorhinolaryngological infections declined from spring [*n*: 87, CI: 70; 105] to summer 2020 [*n*: 61; CI: 47; 77] and increased again in autumn 2020 [*n*: 81; CI: 64; 98], as COVID-19 restrictions were eased [18]. Notably, there were less infections reported across all seasons and diagnoses for the first year of the pandemic. Contrary to 2019, the numbers declined in winter 2020 [*n*: 57; CI: 42; 73], in accordance with the second government-imposed lockdown from December 2020 to March 2021 [19,20]. Spring, summer and autumn 2021 presented a continuous increase which resulted in a plateau in winter 2021 [*n*: 86; CI: 69; 105]. A sharp increase in otorhinolaryngological infections during the entire year 2022 until winter was observed. Comparing the winter seasons individually, we noted significantly more patients in winter 2022 [*n*: 183; CI: 158; 210] in contrast to previous years (*p* < 0.0001), including the pre-pandemic winter 2019 (Figure 1a).

### 3.2. Peritonsillar Abscesses, Acute Tonsillitis and Acute Mastoiditis Were on the Rise Post-Pandemic

The numbers of both peritonsillar abscesses and acute tonsillitis started high in spring 2019 [*n*: 65/31; CI: 51/21; 81/43] (Figure 1b,c). Whilst the number of peritonsillar abscesses remained almost level across the year 2019, the number of acute tonsillitis was greatly reduced until winter 2019. Acute mastoiditis was strongly influenced by the seasons with high numbers in spring 2019, declining until autumn and rising again in winter 2019 (Figure 1d). In 2020, the number of all three entities followed a similar pattern with a decline from spring to summer, a small increase toward autumn and a decrease toward winter. In 2021, the number of patients with acute mastoiditis and acute tonsillitis rose slowly until autumn 2021, with a decline in winter 2021, corresponding to the second government issued lockdown [19,20]. The peritonsillar abscesses increased in spring 2021 and remained almost level all year long. In the post-pandemic year (2022), the numbers of all three entities rose sharply until winter (Figure 1b–d). Comparing seasons individually, we noted significantly more cases of peritonsillar abscesses in winter 2022 [*n*: 92; CI: 74; 112] (*p* < 0.0001). Similarly, the number of patients with acute tonsillitis was significantly higher in 2022 [*n*: 38; CI: 26; 51] across all seasons except autumn (*p* < 0.0001). Likewise, there was a significant difference in the number of cases of acute mastoiditis in 2022 across all seasons except summer, with winter 2022 [*n*: 25; CI: 15; 36] surpassing all previous years and spring 2022 presenting the least amount of cases.

### 3.3. No Post-Pandemic Spike in Mononucleosis

The number of inpatients with mononucleosis differed widely across all years and seasons. Whilst the cases increased rapidly in spring 2019, with a moderate drop in autumn and a renewed increase towards winter, the number of cases in 2020 and 2021 were almost diametrically opposed. Spring to summer 2020 presented a sharp decline with cases rising again toward autumn and a continuous decrease until summer 2021. This was followed by a rise towards autumn and a sharp decrease in winter 2021. 2022 started with a high number of mononucleosis patients, that declined continuously until autumn and leveled in winter 2022. There were no significant differences between seasonal groups (Figure 1e).

### 3.4. The Duration of Hospitalization Was Shorter in 2022

Comparing the length of hospitalization per season across all diagnoses and years, we noted shorter stays in all seasons in 2022 but the difference was only significant (*p* = 0.002) in summer. There were no significant differences in hospitalization, neither in spring (*p* = 0.35), nor in autumn (*p* = 0.16) or winter (*p* = 0.06) (Figure 1f).

### 3.5. Median Age Did Not Change Pre- versus Post-Pandemic

The median age of our patients with regard to their diagnosis group (peritonsillar abscess, acute tonsillitis, mononucleosis and acute mastoiditis) remained unchanged over the years 2019–2023 (*p* = 0.18). Comparing winter 2022 with the previous winters, we found that neither the peritonsillar abscess (*p* = 0.27), nor acute tonsillitis (*p* = 0.44), mononucleosis (*p* = 0.12) or acute mastoiditis (*p* = 0.61) showed a significant shift in age after the COVID-19 pandemic.

However, we observed significant age differences when comparing all the four above-mentioned diagnosis groups against the entire cohort (*p* < 0.0001). The median age of the entire cohort was 29 years. With a median age of 14 years, patients with acute mastoiditis were the youngest group, followed by mononucleosis with 20 years, acute tonsillitis with 26 years and peritonsillar abscess with 33 years.

## 4. Discussion

Our study demonstrates a sharp increase in inpatients with otorhinolaryngological infectious diseases in the post-pandemic winter 2022 surpassing pre-pandemic levels. Interestingly, the seasonal fluctuations in case numbers have not only been impacted during the COVID-19 pandemic, but continue to differ from pre-pandemic patterns in 2022.

Our data are in accordance with several other disrupted infectious diseases, such as an unusual increase in cases of tonsillitis in the United Kingdom in 2022 [14]. Denmark reported a 1.7-fold increase in RSV-associated hospital admissions for pediatric patients in 2022 compared to four pre-pandemic seasons [21]. Moreover, the seasonality was disturbed as the Danish RSV epidemic started early in August instead of the usually reported begin in November [22]. Similar reports about the out-of-season surge in RSV for adults and children from multiple countries in the Northern Hemisphere in 2022 underscore this finding [23,24]. Furthermore, multiple pediatric hospitals in Ireland noted increased cases of invasive bacterial disease like *Streptococcus pneumoniae*, *Streptococcus pyogenes*, *Haemophilus influenzae* or *Neisseria meningitides* with abscesses in head, neck or chest in winter 2022 compared to previous years [25]. Loosen et al. reported a dramatic increase in the incidence of upper respiratory tract infections in adult and pediatric outpatients in Germany in 2022 compared to the pre-pandemic year 2019 [26].

The COVID-19 incidence stagnated in Germany in winter 2022 and the majority of government-implemented non-pharmaceutical interventions expired by March 2023 [27,28]. Despite rigorous testing according to local hospital policies, a few of our patients may have had undetected simultaneous COVID-19 infections, which could have exacerbated their symptoms and led to increased hospitalizations.

So far, there are few reports on the development of otorhinolaryngological infections in post-pandemic times. The incidence of pediatric otitis, tonsillitis and upper respiratory infectious diseases was found to be higher post pandemic, which is in accordance with our data [29]. Torres-García et al. reported reduced admission rates and complications derived from acute otitis media in pediatric otorhinolaryngological patients until March 2021 during the COVID-19 pandemic which is in line with our finding of decreased admissions of patients with acute mastoiditis in 2021 [30]. A reduction in admissions for adult otorhinolaryngological infections was described in the United Kingdom during the pandemic [31]. Galli et al. showed a significant increase in otological infections that required surgical intervention due to beta-hemolytic group A streptococcal infections post-pandemic [32]. In our study, the number of cases with mononucleosis did not follow the seasonal changes or rise above pre-pandemic levels in 2022. McBride et al. reported a reduced incidence of mononucleosis in 2020, but since we investigated the number of hospital admissions rather than the incidence, further studies are needed to truly evaluate the impact of the COVID-19 pandemic on mononucleosis [33]. Data on otorhinolaryngological infections in the post-pandemic year 2022 and beyond, especially including adult patients, could help otorhinolaryngology departments to allocate resources to meet the changing demand.

The reason for these epidemiological changes in infections remains unclear. The concept of so-called “immunity debt”, proposed by the Pediatric Infectious Disease Group among others, offers a possible explanation for the post-pandemic rise in otorhinolaryngological infections [3]. Decreased exposure to common pathogens may result in changes in the development of adaptive immunity or alter the response of the innate immune system [34]. However, altered vaccination programs with diminished and delayed coverage are also thought to have an impact [2,34]. Additionally, healthcare service utilization was heavily impacted by the COVID-19 pandemic [2]. Non-pharmaceutical interventions such as personal hygiene, face masks and social distancing are thought to have reduced the number of admissions for respiratory infections and infectious contacts by changes in population behavior [3,35]. Another possible explanation is the termination of non-pharmaceutical intervention itself which was implemented by the government in March 2022 and correlates with our finding of increased numbers of inpatients with otorhinolaryngological infectious diseases over the course the year 2022 [36].

Interestingly, the median age of patients within their diagnosis group did not increase significantly during or after the pandemic but remained similar to the pre-pandemic age. This suggests that the onset of infection was not significantly delayed by non-pharmaceutical interventions.

García-Callejo et al. reported a significantly longer duration of hospitalization of patients with a peritonsillar abscess in 2020 compared to previous years [37]. This contrasts our findings as we observed no significant difference. However, the duration of hospitalization was shorter in 2022 compared to previous years. Whether this is a surrogate for a milder course of disease or an expression of the post-pandemic lack of resources cannot be clarified in this study and needs further attention.

Due to our geographically isolated location as the only tertiary care department of otorhinolaryngology—head and neck surgery in the region we collected a robust data set with relatively high case numbers. However, in order to better analyze the impact of the COVID-19 pandemic on otorhinolaryngological infections, it would provide better insight to assess not only the case numbers but also the severity of clinical courses. The situation of outpatient treatment for otorhinolaryngological infections is not taken into account in this study. Both could provide valuable information if the need for inpatient treatment is growing and whether certain diseases should increasingly be treated on an outpatient basis in a time of scarce resources of healthcare systems. Moreover, prospective studies should be carried out to evaluate coming years providing a scientific background for future public health policies.

In conclusion, the impact of the COVID-19 pandemic on otorhinolaryngological infections has lasted beyond social distancing measures and requires further investigation.

## Data Availability

Ethical approvement does not include supplementing the raw data.
